# Variants in the 15q24/25 Locus Associate with Lung Function Decline in Active Smokers

**DOI:** 10.1371/journal.pone.0053219

**Published:** 2013-01-18

**Authors:** Firdaus A. A. Mohamed Hoesein, Els Wauters, Wim Janssens, Harry J. M. Groen, Joanna Smolonska, Cisca Wijmenga, Dirkje S. Postma, H. Marike Boezen, Pim A. De Jong, Marc Decramer, Jan-Willem J. Lammers, Diether Lambrechts, Pieter Zanen

**Affiliations:** 1 Division of Heart and Lungs, Department of Respiratory Medicine, University Medical Center, Utrecht, The Netherlands; 2 Respiratory Division, University Hospital Gasthuisberg, KU Leuven, Leuven, Belgium; 3 Vesalius Research Center, VIB, Leuven, Belgium; 4 Vesalius Research Center, KU Leuven, Leuven, Belgium; 5 Department of Pulmonology, University Medical Center Groningen, University of Groningen, Groningen, The Netherlands; 6 Department of Genetics, University Medical Center Groningen, University of Groningen, Groningen, The Netherlands; 7 Department of Epidemiology, University Medical Center Groningen, University of Groningen, Groningen, The Netherlands; 8 Department of Radiology, University Medical Center, Utrecht, The Netherlands; Johns Hopkins School of Medicine, United States of America

## Abstract

Genetic variation in nicotinic acetylcholine receptor subunit genes (*nAChR*s) is associated with lung function level and chronic obstructive pulmonary disease (COPD). It is unknown whether these variants also predispose to an accelerated lung function decline. We investigated the association of *nAChR* susceptibility variants with lung function decline and COPD severity. The rs1051730 and rs8034191 variants were genotyped in a population-based cohort of 1,226 heavy smokers (COPACETIC) and in an independent cohort of 883 heavy smokers, of which 653 with COPD of varying severity (LEUVEN). Participants underwent pulmonary function tests at baseline. Lung function decline was assessed over a median follow-up of 3 years in COPACETIC. Current smokers homozygous for the rs1051730 A-allele or rs8034191 G-allele had significantly greater FEV_1_/FVC decline than homozygous carriers of wild-type alleles (3.3% and 4.3%, p = 0.026 and p = 0.009, respectively). In the LEUVEN cohort, rs1051730 AA-carriers and rs8034191 GG-carriers had a two-fold increased risk to suffer from COPD GOLD IV (OR 2.29, 95% confidence interval [CI] = 1.11–4.75; p = 0.025 and OR = 2.42, 95% [CI] = 1.18–4.95; p = 0.016, respectively). The same risk alleles conferred, respectively, a five- and four-fold increased risk to be referred for lung transplantation because of end-stage COPD (OR = 5.0, 95% [CI] = 1.68–14.89; p = 0.004 and OR = 4.06, 95% [CI] = 1.39–11.88; p = 0.010). In Europeans, variants in *nAChRs* associate with an accelerated lung function decline in current smokers and with clinically relevant COPD.

## Introduction

Chronic obstructive pulmonary disease (COPD) is characterized by progressive airflow limitation as reflected by an accelerated decline in forced expiratory volume in one second (FEV_1_) [1]. Unfortunately, symptoms in COPD often only become apparent after a significant loss of lung function, thereby delaying diagnosis and negatively influencing prognosis. Smoking is by far the most important risk factor for COPD [1]. Although cigarette smokers have a larger FEV_1_ decline than non-smokers, only 10–20% of them develop COPD [2]. Additional susceptibility factors predictive of an accelerated lung function decline thus need to be identified, allowing the implementation of appropriate strategies to prevent patients from developing severe COPD.

Genetic predisposition to COPD development is actively being explored [3]. For many years, uncommon mutations in the gene encoding the alpha 1-antitrypsin protein represented the only established genetic risk factor for severe, early-onset COPD [4]. Recently, several genome-wide association studies (GWAS) identified multiple novel genetic risk factors [5]–[7]. One of these at-risk loci is located on chromosome 15q24/25 in a region that contains the nicotinic acetylcholine receptor subunit genes (*nAChR*s) [7]. Interestingly, *nAChR* variants have also been associated with smoking addiction [8]–[11], peripheral arterial disease [12], lung cancer [12]–[14] and emphysema [15] indicating that the *nAChR* locus is implicated in the development of smoking-related conditions.

Although it is widely accepted that COPD results from a progressive decline in lung function [16], it has not yet been established whether *nAChR* variants predisposing to COPD are also associated with an accelerated lung function decline. In addition, it is currently unclear how smoking behavior may interact with this genetic locus on disease progression. We therefore assessed whether rs1051730 and rs8034191, two variants in the *nAChR* locus, were associated with the decline in FEV_1_, FEV_1_/FVC and MEF_50_ over 3 years in subjects from a large population-based cohort (COPACETIC, n = 1,226). As proof of concept, we additionally assessed the predictive value of our findings in an independent group of heavy smokers (LEUVEN), consisting of healthy smokers (n = 230) and patients diagnosed with COPD of varying stages of severity (GOLD I–IV, n = 653).

## Materials and Methods

### Study subjects

The COPACETIC cohort included 1,226 Dutch participants from the NELSON lung cancer screening trial recruited by the University Medical Centers of Groningen and Utrecht [17]. In brief, all participants were male heavy smokers (≥20 or more pack-years), aged between 50–75 years and fit enough to undergo surgery.

The LEUVEN cohort included 366 Belgian participants of the Dutch-Belgian randomized lung cancer screening trial (NELSON) who were recruited from the general population of 14 municipalities around LEUVEN and who were not previously diagnosed with COPD. In addition, 517 COPD patients were included at the outpatient clinic of the University Hospital Gasthuisberg in Leuven. Of these 517 subjects, 123 were listed for lung transplantation because of disabling end-stage COPD [18]. The 883 LEUVEN participants were all heavy smokers, matched for smoking history (>15 pack-years) and age (>50 years). All LEUVEN participants self-declared Belgian-Flemish ethnicity for three generations [19].

Participants from both COPACETIC and LEUVEN, all of Caucasian ancestry, provided written informed consent. The complete inclusion criteria for COPACETIC and LEUVEN are described in detail in Material S1.

### Ethics statement

The UZ LEUVEN Medical Ethics Committee (Leuven, Belgium) and the University Medical Center Utrecht Institutional Review Board both approved the study protocol.

### Pulmonary function testing

All participants underwent pulmonary function tests with standardized equipment according to the American Thoracic Society (ATS) and European Respiratory Society (ERS) guidelines [20]. To investigate the influence of genetic variability on decline in FEV_1_ and FEV_1_/FVC, pulmonary function tests were performed at inclusion and after three years of follow-up in COPACETIC. We also evaluated the decline in MEF_50_, which represents the maximal expiratory flow at 50% FVC and may reflect changes in the smaller peripheral airways [20]. Bronchial obstruction was established as a post-bronchodilator FEV_1_/FVC ratio of <0.70 in LEUVEN and as a pre-bronchodilator FEV_1_/FVC ratio of <0.70 in COPACETIC [1]. Severity was staged by FEV_1_ expressed as % predicted according to the Global Initiative for Chronic Obstructive Lung Disease (GOLD) classification [1].

### Genotyping

Two variants in the 15q24/25 locus, rs1051730 and rs8034191, were selected based on previous GWAS for COPD and lung cancer [7], [12]–[14]. Genotyping was conducted in analogy to a previously published study, in which we established an association between a single SNP, rs1051730, and the risk of COPD and emphysema [15]. In particular, COPACETIC genotypes were extracted from Human610-Quad BeadChip data generated in the COPACETIC GWAS (Illumina Inc., San Diego, CA, USA), whereas LEUVEN participants were genotyped in a blinded manner using iPLEX technology on a MassARRAY Compact Analyser (Sequenom Inc., San Diego, CA, USA). A more detailed description is given in Material S2.

### Statistical Analysis

Means and standard deviations (SD) were calculated for normally distributed variables, and medians and interquartile ranges for non-normally distributed variables. Chi-square tests were used to test for differences in demographics and nicotine-addiction related variables between genotypes. Multivariate regression analyses correcting for age, smoking status and study center were performed to assess the association of the genotypes with smoking behavior. In both cohorts (COPACETIC and LEUVEN), all analyses for both variants were performed without assuming a specific model of inheritance, i.e., by conducting a genotypic test.

In COPACETIC, multiple regression analyses were performed to test the association of rs1051730 and rs8034191 with changes in FEV_1_/FVC, FEV_1_ and MEF_50_ over time. To differentiate between genetic variability in lung function decline and a genetically determined lower lung function level, adjustments for baseline FEV_1_, FEV_1_/FVC and MEF_50_ were made. In particular, adjustments were made for study center, age, height, smoking status (current/former smoker), pack-years, years in study and baseline FEV_1_/FVC, FEV_1_, MEF_50_. To test whether the effect of genotype differed according to smoking status, we also inserted an interaction between genotype and smoking status. We particularly assessed the interaction with smoking status since this variable is the most important differentiating smoking-related variable in our study population of heavy smokers (with >20 pack-years of smoking at inclusion). Change in lung function was calculated by subtraction of the baseline lung function values from the predicted follow-up values derived from the multiple regression analyses. In addition, we calculated the observed power to discover a significant association of genotypes, smoking status (current versus former) and the genotype*smoking status interaction term with lung function decline. The methods and results of power calculations are provided in Material S3. In COPACETIC, the p-value threshold for significance adjusted for testing three clinical variables (decline in FEV_1_/FVC, FEV_1_ and MEF_50_) using the Bonferroni correction method, resulting in a significance threshold of p<0.0167.

In LEUVEN, the relationship between rs1051730 or rs8034191 genotypes and GOLD stage was assessed by chi-square analysis. The association between genotypes and the risk of developing very severe COPD (GOLD IV) was confirmed via multinomial logistic regression analysis, while correcting for age, height, sex, pack-years and years-quit for former smokers. In addition, given the extensive heterogeneity in degree of functional impairment among COPD patients, even with the same level of airflow obstruction, we classified the LEUVEN subjects in three clinical subgroups: *(i)* asymptomatic smokers, defined as heavy smokers that do not report respiratory symptoms and are therefore not diagnosed with COPD, *(ii)* ambulatory COPD patients, defined as patients with well-established COPD routinely attending the outpatient pulmonary clinic, and *(iii)* patients listed for lung transplantation because of the severe repercussion of COPD for their daily life activities and life expectancy (<18 months). Differences between these subgroups were assessed using a Pearson's chi-square analysis. A multinomial logistic regression analysis to assess the probability of belonging to any of these clinical subgroups in function of genotypes was performed, while correcting for age, height, sex, pack-years and years-quit for former smokers. P-values<0.05 were considered significant in the LEUVEN cohort. All statistical analyses were performed using SPSS 18 for Windows (SPSS, Chicago, Illinois, USA).

## Results

### Population Characteristics

In total, 1,226 participants were included in COPACETIC and 883 participants in LEUVEN. Demographics, smoking history, pulmonary function measurements and the prevalence of COPD is shown for all COPACETIC and LEUVEN participants in Table 1. There were no significant differences in pack-years between former and current smokers (p = 0.188 and p = 0.309 for COPACETIC and LEUVEN, respectively; data not shown). Baseline characteristics for the LEUVEN clinical subgroups are shown in Table 2.

**Table 1:** Characteristics for COPACATIC and LEUVEN at baseline and after three-year follow-up for COPACETIC.

	COPACETIC		LEUVEN
	Baseline (n = 1,226)	Follow-up (n = 1,226)	(n = 883)
**Demographics**			
Age, mean (SD), yr	59.7 (5.3)	62.7 (5.4)	63.9 (8.0)
Male sex, no. (%)	1,226 (100)	1,226 (100)	656 (74.0)
Height, mean (SD), cm	177.5 (6.5)	178.1 (6.3)	169.6 (8.9)
**Smoking**			
Pack-year history, mean (SD), yr	40.7 (17.4)	N/A	47.4 (24.8)
Current smokers, no. (%)	753 (61.4)	N/A	367 (41.3)
Smoked years, mean (SD), yr	39.6 (8.87)	N/A	41.7 (9.0)
Years quit smokers, median (25^th^–75^th^ percentiles)	8.1 (3.0–9.0)	N/A	1.0 (0.0–8.0)
**Pulmonary function tests, mean (SD)**			
FEV_1_, L	3.33 (0.73)	3.14 (0.72)	1.92 (1.08)
FEV_1_, % predicted	96.5 (18.4)	93.7 (18.8)	65.4 (32.7)
FVC, L	4.70 (0.79)	4.61 (0.81)	3.36 (1.13)
FVC, % predicted	107.1 (14.6)	106.7 (15.3)	91.9 (24.3)
MEF_50_, L/s	3.08 (1.45)	2.76 (1.38)	1.52 (1.50)
FEV_1_/FVC ratio	0.71 (0.10)	0.68 (0.10)	0.54 (0.18)
**COPD severity, no. (%)**			
No Obstruction	682 (55.6)	557 (45.4)	230 (26.0)
GOLD class I	357 (29.1)	430 (35.1)	123 (13.9)
GOLD class II	162 (13.2)	210 (17.1)	183 (20.7)
GOLD class III	24 (2.0)	23 (1.9)	188 (21.2)
GOLD class IV	0	5 (0.4)	159 (18.0)

FEV_1_: forced expiratory volume in one second; FVC: forced vital capacity; COPD: chronic obstructive pulmonary disease; GOLD: global initiative for chronic obstructive lung disease; N/A: non-applicable. Percentages are column percentages. Of the LEUVEN participants, 653 subjects were diagnosed with COPD (FEV_1_/FVC<0.70).

**Table 2:** Baseline Characteristics for LEUVEN participants stratified for the clinical impact of airflow obstruction.

	Asymptomatic smokers	Ambulatory COPD patients	End-stage COPD patients	p-value
	(n = 366)	(n = 394)	(n = 123)	
**Demographics**				
Age, mean (SD), yr	62.3 (5.71)	67.6 (8.67)	57.2 (4.69)	<0.001
Male sex, no. (%)	286 (78.1)	305 (77.4)	62 (50.4)	<0.001
Height, mean (SD), cm	172.0 (8.9)	168.6 (8.4)	165.0 (8.5)	<0.001
**Smoking**				
Pack-years history, mean (SD), yr	45.8 (21.8)	52.5 (27.3)	35.5 (19.8)	<0.001
Current smokers, no. (%)	203 (55.8)	157 (40.5)	5 (15.6)	<0.001
Smoked years, mean (SD), yr	40.6 (7.0)	43.2 (10.4)	36.1 (8.2)	<0.001
Years quit smokers, median (25^th^–75^th^ percentiles)	0.0 (0.0–7.0)	2.0 (0.0–9.0)	3.0 (1.0–6.0)	<0.001
**Pulmonary function tests, mean (SD)**				
FEV_1_, L	2.91 (0.74)	1.38 (0.63)	0.70 (0.30)	<0.001
FEV_1_, % predicted	96.4 (18.3)	49.3 (20.1)	25.5 (9.9)	<0.001
FEV_1_/FVC ratio	0.70 (0.09)	0.45 (0.13)	0.33 (0.08)	<0.001
**COPD severity, no. (%)**				
No Obstruction	218 (59.6)	11 (2.8)	0 (0)	<0.001
GOLD class I	96 (26.2)	27 (6.9)	1 (0.8)	
GOLD class II	46 (12.6)	134 (34.0)	3 (2. 4)	
GOLD class III	6 (1.6)	157 (39.8)	25 (20.3)	
GOLD class IV	0 (0)	65 (16.5)	94 (76.4)	

Abbreviations are the same as in Table 1. Percentages are column percentages. The 366 asymptomatic smokers are population-based participants of the Dutch-Belgian lung cancer screening trial (NELSON). None of them were previously diagnosed with COPD. However, 148 subjects (40.4%) were found to have an obstructive lung function (based on FEV_1_/FVC<0.70) at inclusion. Eleven subjects (2%), that were followed-up at the outpatient clinic because of symptoms compatible with COPD, did not fulfill the criterion of COPD (FEV_1_/FVC>0.70). Five patients with end-stage COPD were not actively listed for lung transplantation because of their current smoking status at inclusion.

In COPACETIC, genotyping for both rs1051730 and rs8034191 succeeded in 99% of participants. In LEUVEN genotyping for rs1051730 and rs8034191 succeeded in 99% and 100%, respectively. Genotype frequencies were similar as observed in the HAPMAP databases and other studies [7], [21]. There was strong linkage disequilibrium between rs1051730 and rs8034191 in both cohorts (r^2^ = 0.86 for COPACETIC and r^2^ = 0.88 for LEUVEN). Baseline characteristics for LEUVEN and COPACETIC participants according to rs1051730 and rs8034191 genotypes are shown in Table 3 and Table 4.

**Table 3:** Baseline characteristics for the COPACETIC cohort according to rs1051730 and rs8034191 genotypes.

	rs1051730				rs8034191			
	AA(n = 134)	GA(n = 562)	GG(n = 530)	p-value	GG(n = 133)	GA(n = 560)	AA(n = 531)	p-value
**Demographics**								
Age, mean (SD), yr	59.6 (6.0)	59.6 (5.3)	59.7 (5.4)	0.952	59.6 (5.7)	59.6 (5.2)	59.8 (5.4.)	0.739
Pack year history, mean (SD), yr	39.3 (15.9)	41.4 (17.4)	40.2 (17.7)	0.301	39.7 (17.4)	41.6 (17.6)	39.9 (17.2)	0.209
Current smokers, no (%)	76 (56.7%)	333 (59.3%)	344 (64.9%)	0.093	75 (56.4%)	341 (60.9%)	337 (63.5%)	0.298
**Years quit smoking % (no)**				0.307				0.356
0	57.8% (78)	59.4% (337)	64.8% (346)		57.0% (75)	60.9% (344)	63.4% (339)	
<1	7.4% (10)	6.4% (35)	7.8% (41)		6.7% (9)	6.5% (35)	7.9 5 (42)	
1–5	18.5% (24)	18.5% (102)	14.3% (74)		19.3% (25)	17.5% (97)	15.2% (79)	
>5	16.3% (22)	15.7% (88)	13.1% (69)		17.0% (23)	15.2% (84)	13.5% (71)	
**Age started smoking % (no)**				0.491				0.397
<14 years	14.9% (20)	19.2% (118)	17.4% (92)		16.5% (22)	18.8% (111)	16% (87)	
15–19 years	70.2% (94)	65.8% (395)	67.7% (359)		67.0% (91)	65.7% (378)	68.4% (368)	
>20	14.9% (20)	15.0% (48)	14.9% (79)		16.5% (22)	11.8% (71)	13.7% (76)	
**Pulmonary function tests, mean (SD)**								
FEV_1_, L	3.26 (0.76)	3.29 (0.73)	3.41 (0.72)	0.013	3.24 (0.74)	3.29 (0.74)	3.41 (0.71)	0.008
FEV_1_, % predicted	94.4 (19.5)	95.1 (18.5)	98.6 (17.9)	0.003	94.1 (18.8)	95.2 (19.0)	98.6 (17.4)	0.002
FEV_1_/FVC ratio	0.70 (0.10)	0.71 (0.10)	0.72 (0.107)	0.002	0.71 (0.10)	0.70 (0.11)	0.72 (0.10)	0.002
MEF_50_, L/s	2.97 (1.44)	2.95 (1.39)	3.25 (1.50)	0.002	2.94 (1.38)	2.96 (1.44)	3.24 (1.47)	0.003
**COPD severity, no. (%)**				0.004				0.010
No Obstruction	67 (50%)	291 (51.6%)	326 (61.3%)		71 (52.2%)	286 (51.2%)	326 (61.2%)	
GOLD class I	38 (28.4%)	181 (32.2%)	138 (26.0%)		39 (28.4%)	176 (31.4%)	143 (26.9%)	
GOLD class II	27 (20.1)	74 73 (13.2%)	61 (11.5%)		24 (17.9%)	81 (14.4%)	57 (10.7%)	
GOLD class III	2 (1.5%)	17 17 (3.0%)	5 (0.9%)		2 (1.5%)	17 (3.0%)	5 (0.9%)	

FEV_1_: forced expiratory volume in one second; FVC: forced vital capacity; MEF_50_ maximum expiratory flow when 50% of the FVC has been exhaled; GOLD: global initiative for chronic obstructive lung disease. Percentages are column percentages. Genotyping succeeded in 1226 (100%) and 1224 (99.8%) COPACETIC participants, respectively for rs1051730 and rs8034191.

**Table 4:** Baseline characteristics for the LEUVEN group according to rs1051730 and rs8034191 genotypes.

	rs1051730				rs81034191			
	AA	AG	GG	P-value	GG	AG	AA	P-value
**Demographics**								
Age	63.9 (7.8)	63.4 (8.0)	64.7 (8.0)	0.086	63.5 (7.9)	63.6 (8.0)	64.6 (8.0)	0.234
Pack-years history, mean (SD), yr	45.5 (21.6)	49.6 (26.7)	45.9 (23.9)	0.080	46.2 (22.2)	49.4 (26.6)	45.7 (23.5)	0.103
Current smokers, no (%)	44 (38.3)	170 (47.2)	147 (48.8)	0.144	52 (41.3)	167 (45.5)	146 (50.2)	0.211
**Pulmonary function tests, mean (SD)**								
FEV_1_, L, post	1.74 (1.04)	1.93 (1.09)	1.98 (1.07)	0.088	1.77 (1.06)	1.93 (1.08)	1.98 (1.08)	0.146
FEV_1_, % predicted, post	59.8 (31.6)	65.5 (32.6)	68.1 (33.1)	0.044	60.7 (32.6)	65.6 (32.0)	67.5 (33.4)	0.111
FEV_1_/FVC ratio,	0.51 (0.18)	0.54 (0.18)	0.55 (0.18)	0.054	0.51 (0.18)	0.54 (0.18)	0.55 (0.18)	0.045
**COPD severity, no. (%)**				0.444				0.385
No bronchial obstruction	30 (13.1)	106 (46.3)	93 (40.6)		31 (13.5)	109 (47.4)	90 (39.1)	
GOLD class I	18 (14.8)	51 (41.8)	53 (43.4)		22 (17.9)	52 (42.3)	49 (39.8)	
GOLD class II	27 (15.0)	89 (49.4)	64 (35.6)		28 (15.3)	93 (50.8)	62 (33.9)	
GOLD class III	27 (14.4)	85 (45.5)	75 (40.1)		29 (15.4)	86 (45.7)	73 (38.8)	
GOLD class IV	33 (21.2)	71 (45.5)	52 (33.3)		36 (22.6)	69 (43.4)	54 (34.0)	
**Clinical Category, no. (%)**				0.018				0.067
Asymptomatic smokers	43 (11.8)	175 (48.2)	145 (39.9)		48 (13.1)	176 (48.1)	142 (38.8)	
Ambulatory COPD patients	62 (15.9)	176 (45.1)	152 (39.0)		68 (17.3)	182 (46.2)	144 (36.5)	
Patients with end-stage COPD	30 (24.8)	51 (42.1)	40 (33.1)		30 (24.4)	51 (41.5)	42 (34.1)	

Genotyping succeeded in 874 (99%) and 883 (100%) LEUVEN participants, respectively for rs1051730 and rs8034191.

### Association of nAChR variants with nicotine addiction-related variables

To assess whether rs1051730 and rs8034191 were associated with nicotine addiction, we tested for association with pack-years, smoking status (current/former smoker), quit-years in former smokers (>5, 1–5, <1 years) and age-started smoking (<14 years, 15–19 years, >20 years) in COPACETIC. No significant associations were found (Table 3). Moreover, multivariate regression analysis, correcting for age, smoking status and study center, showed no significant effect of rs1051730 and rs8034191 on pack-years smoked (p = 0.327 and 0.258, respectively).

Likewise, no significant association between rs1051730 and rs8034191 genotypes, and smoking status (current/former) or pack-years was found in LEUVEN (respectively, p = 0.144 and p = 0.086 for rs1051730, p = 0.211 and P = 0.103 for rs8034191; Table 4). Multivariate regression analysis, correcting for age, gender and smoking status, showed no significant effects for rs1051730 and rs8034191 on pack-years smoked (p = 0.133 and 0.140, respectively; data not shown).

### Association of nAChR variants with lung function decline in COPACETIC

Lung function measurements at baseline and after a median follow-up time of 3 years (interquartile range 2.9–3.1) are provided in Table 1. Mean FEV_1_/FVC at baseline was 70.9±10.2% and 68.0±9.6% at follow-up, representing a mean decrease of 2.9%. Mean decrease of FEV_1_ and MEF_50_ were 193 mL and 322 mL/s, respectively. Baseline lung function measurements stratified for rs1051730 and rs8034191 are provided in Table 3.

In a multivariate regression analysis to test the association of both genotypes with lung function decline, the interaction between rs1051730 and smoking status was significant (p = 0.015 for interaction term). This indicates that the effect of rs1051730 genotypes differed between current and former smokers. Current smokers homozygous for the rs1051730 A-allele had a more pronounced decline in FEV_1_/FVC compared to current smokers homozygous for the G-allele (4.3% and 3.3%, p = 0.026, Table 5). In contrast, former smokers carrying the AA genotype had no such stronger decline in FEV_1_/FVC compared to former smoking GG-carriers (1.5% and 2.4%, p = 0.317, Table 5).

**Table 5:** Lung function decline in COPACETIC according to rs1051730 and rs8034191 genotypes.

		rs1051730					rs8034191		
		FEV1/FVC [%]	FEV1 [mL]	MEF50 [mL/s]			FEV1/FVC [%]	FEV1 [mL]	MEF50 [mL/s]
Current smoker	AA	4.30 (0.82)#	229 (57)	491 (220)	Current smoker	AA	2.72 (0.87)	246 (52)	377 (232)
	AG	2.97 (0.97)	203 (56)	316 (245)		AG	2.87 (0.95)	200 (62)	315 (252)
	GG	3.30 (0.88)	243 (51)	393 (237)		GG	3.31 (0.85)*	232 (56)	485 (211)*
Former smoker	AA	1.53 (1.07)	140 (60)	167 (250)	Former smoker	AA	2.44 (0.89)	171 (58)	271(253)
	AG	2.47 (0.99)	180 (56)	289 (233)		AG	2.48 (1.00)	180 (58)	281 (236)
	GG	2.41 (0.90)	167 (57)	253 (259)		GG	1.39 (1.06)	130 (65)	146 (250)

# significant compared to the GG genotype for rs1051730 (p = 0.026).

* significant compared to AA genotype for rs8034191 (p = 0.009 and p = 0.017, respectively for FEV_1_/FVC and MEF_50_ decline).

Similar results were obtained for the interaction between rs8034191 and smoking status (p = 0.002 for interaction term). Current smokers homozygous for the rs8034191 G-allele showed a significantly stronger decline of the FEV_1_/FVC compared to current smoking participants homozygous for A-alleles (3.3% and 2.7%, p = 0.009). For heterozygotes this decline was 2.9%. As for rs1051730, former smokers homozygous for the rs8034191 G-allele had no significant additional FEV_1_/FVC decline compared to former smoker homozygous for the A-allele (1.4% and 2.4%, p = 0.465). When performing similar analyses for FEV_1_ neither of the two SNPs was significantly associated with a lower FEV_1_ at follow-up (p = 0.964 and 0.857, respectively) and none of the interactions between SNPs and smoking status were significant (p = 0.203 and 0.107, respectively).

When analyzing MEF_50_, a similar effect as observed with FEV_1_/FVC was noted (p = 0.047 and 0.036 for rs105170 or rs8034191, respectively). Smokers homozygous for the rs1051730 A-allele had a 491 mL/s decline in MEF_50_ compared to a 393 mL/s in GG-carriers (p = 0.083). A similar pattern was found for smokers carrying the rs8034191 GG genotype (485 mL/s decline compared to 377 mL/s, p = 0.017) in current smokers. In heterozygotes the decline was 314 mL/s. For both SNPs there were no significant differences in former smokers (167 mL/s and 253 mL/s, p = 0.280 and 146 mL/s and 271 mL/s, p = 0.188, respectively for rs1051730 and rs8034191).

To additionally demonstrate that the association of rs1051730 and rs8034191 with decline in FEV_1_/FVC and MEF_50_ in active smokers was independent of baseline lung function level, we inserted the interaction term baseline FEV_1_/FVC*genotype or MEF_50_*genotype as a covariate in the regression model. As expected, these analyses did not reveal a significant effect for these interaction terms (p = 0.225 and p = 0.310 for FEV_1_/FVC and p = 0.248 and p = 0.172 for MEF_50_, respectively for rs1051730 and rs8034191). The significance values of the interaction terms genotype*lung function parameter (FEV_1_/FVC and MEF_50_) are listed in Table 6.

**Table 6:** Significance values of the interaction terms genotype*lung function parameter in the multiple linear regression model of lung function decline in COPACETIC.

Interaction term	p-value	Interaction term	p-value
rs1051730 * FEV_1_/FVC	0.225	rs8034191* FEV_1_/FVC	0.310
rs1051730 * MEF_50_	0.248	rs8034191* MEF_50_	0.172

### Association between nAChR variants and severity of COPD

To assess the clinical relevance of an accelerated lung function decline due to variation in *nAChR* genes, we studied the association of rs1051730 and rs8034191 with COPD severity and symptoms in an independent group of heavy smokers (LEUVEN). A multinomial logistic regression analysis was performed to assess the association between both genotypes and the risk of developing very severe COPD (GOLD IV). AA-carriers of the rs1051730 genotype had a two-fold increased risk (OR 2.29, 95% confidence interval [CI] = 1.11–4.75; p = 0.025) of suffering from COPD GOLD IV compared to GG-carriers. Likewise, GG-carriers of rs8034191 (compared to AA-carriers) had a two-fold increased risk for GOLD IV versus no COPD (OR 2.42, 95% [CI] = 1.18–4.95; p = 0.016). No association was observed between rs1051730 or rs8034191 and lower GOLD stages (p = 0.837, p = 0.406 and P = 0.933, respectively for the association between rs1051730 and GOLD I, II and III and p = 0.373, p = 0.339 and P = 0.633, respectively for the association between rs8034191 and GOLD I, II and III).

To establish whether the accelerated lung function decline in at-risk smokers also influences the severity of disease presentation, we classified all the LEUVEN subjects in three clinical categories (Table 2). We observed a significant association between rs1051730 and the clinical subgroups (p = 0.018; Table 4). Indeed, carriers of the at-risk AA genotype were twice as frequent in the most severe COPD group (24.8% in patients with end-stage COPD versus 15.9% and 11.8% in ambulatory COPD patients and asymptomatic smokers). Moreover, multinomial logistic regression revealed that (compared to GG) AA-carriers of the rs1051730 genotype exhibited an odds ratio of 5.0 (95% [CI] = 1.68–14.89; p = 0.004) for receiving lung transplantation. Likewise, AA-carriers had a 1.48-fold increased risk of being an ambulatory COPD patient (95% [CI] = 0.90–2.42; p = 0.119). The latter analysis was not significant presumably because some of the asymptomatic heavy smokers in LEUVEN already developed COPD (respectively 12.5% and 1.6% exhibited COPD with GOLD II and III). Similar data were observed for the rs8034191 SNP, as GG-carriers had a 4-fold increased risk of evolving to end-stage COPD with need of lung transplantation (OR = 4.06; 95% [CI] = 1.39–11.88; p = 0.010) and a 1.56-fold increased risk of belonging to the group of ambulatory COPD patients (OR = 1.56; 95% [CI] = 0.97–2.51; p = 0.069).

Given the interaction between both nAChR variants and smoking status in the COPACETIC cohort, we additionally corrected our multinominal logistic regression analysis in the LEUVEN cohort for the interaction term rs1051730*smoking status or rs8034191*smoking status. The interaction was not found to significantly influence the risk of developing COPD GOLD stage IV (OR = 0.24, 95% [CI] = 0.04–1.37; p = 0.108 for rs1051730*smoking status and OR = 0.25, 95% [CI] = 0.05–1.30; p = 0.101 for rs8034191*smoking status). Likewise, the interaction term was not found to significantly influence the severity of disease presentation (OR = 0.47, 95% [CI] = 0.02–10.9; p = 0.637 for rs1051730*smoking status and OR = 0.45, 95% [CI] = 0.02–10.1; p = 0.618 for rs8034191*smoking status).

## Discussion

In the current study, we observed that two common variants in the *nAChR* locus on chromosome 15q24/25 affect FEV_1_/FVC decline in a population-based sample consisting of heavy smokers (COPACETIC). To the best of our knowledge, our study is the first to show an association of the 15q24/25 locus with decline in lung function over time. Importantly, this genotype-associated difference in lung function decline was independent of baseline lung function level, indicating that variants in the *nAChR* locus are not merely associated with an inherited lower lung function level, but also with an accelerated decline in lung function. Remarkably, no such effect was observed in former smokers. Based on power calculations, which revealed that our study had respectively 81.9% and 88.0% power for rs1051730 and rs8034191 to detect a significant difference in FEV_1_/FVC decline in former smokers (Material S3), we believe that the absence of genetic variability in lung function decline in former smokers is a true negative finding. Importantly, we also established that the accelerated lung function decline in rs1051730 AA-carriers and rs8034191 GG-carriers may be relevant for clinical practice. For instance, compared to GG-carriers, homozygotes for the rs1051730 A-allele showed a two-fold increased risk (OR = 2.29) to be diagnosed with very severe COPD GOLD IV and exhibited an OR of 5.0 to have end-stage COPD with need for lung transplantation [22]. Likewise, GG-carriers of the rs8034191 genotype had a two-fold increased risk (OR = 2.42) to have GOLD stage IV disease and a four-fold increased risk (OR = 4.06) to be in need of lung transplantation compared to AA-carriers. Overall, these data suggest that current smokers carrying two copies of the at-risk alleles in the nAChR genes have an accelerated lung function decline, which may reflect susceptibility towards the development of severe COPD. The association between the same at-risk genotypes and very severe COPD supports this hypothesis. Future experiments are now warranted to investigate the association between genetic variation in the nAChR genes, lung function decline and incident COPD.

The observed associations may be biased for differences in smoking behavior between the *nAChR* at-risk and wild type genotypes. Indeed, recent GWA studies have indicated that the *nAChR* risk variants increase the risk of smoking addiction, presumably by mediating addictive effects in the brain, and promote more intense smoking, as reflected by a higher level of tobacco-specific nitrosamines per cigarette smoked in homozygous carriers of the at-risk alleles [23], [24]. However, we did not find an association between *nAChR* variants and nicotine addiction related variables in our study population. Furthermore, the observed association between *nAChR* variants and lung function decline withstood correction for smoking status (current versus former smokers), years-quit smoking and pack-years smoked. Nicotinic acetylcholine receptors (*nAChR*s), which are encoded by the *CHRNA* genes, are also widely expressed on airway epithelial cells and immune cells, such as macrophages, and their role in mediating inflammatory processes has been established [25], [26]. It is therefore possible that *nAChR*s directly affect lung parenchyma and that genetic variation modulates the inflammatory response upon stimulation of *nAChR*s by its agonists, thereby determining the extent of lung function decline. To completely discriminate between a direct and indirect effect, the relationship between the 15q24/25 locus and COPD could be studied among never-smokers. However, this approach is difficult given the very small number of never-smoking COPD patients. Alternatively, new statistical approaches could be applied, such as for instance mediation analysis. By use of this technique, Wang et al. established a direct association between rs1051730 and COPD risk (P = 0.046), but also an indirect effect mediated by the variability in smoking behavior according to rs1051730 genotypes (0.006) [27]. These findings are similar to our results: we did not establish an association between the 15q24/25 locus and nicotine addiction related variables. However, we demonstrated that rs1051730 affects lung function decline only in the group of active smokers, suggesting that this SNP exerts a mediating, but not a causal influence of smoking behavior. The hypothesis of a dual association (direct and indirect via smoking behavior) between 15q24/25, lung function decline and COPD should be investigated in future studies.

Moreover, since our study was performed in subjects from Caucasian ancestry, we cannot make any assumptions on the association between nAChR variants and lung function parameters or COPD in other ethnic groups. In a recent study in a Chinese Han population, no significant association was found between rs1051730 or rs8034191 and COPD in either former or current smokers. On the other hand, rs8034191 and rs1051730 were both associated with FEV1% predicted and FEV1/FVC in COPD cases [28].

**Figure 1:**
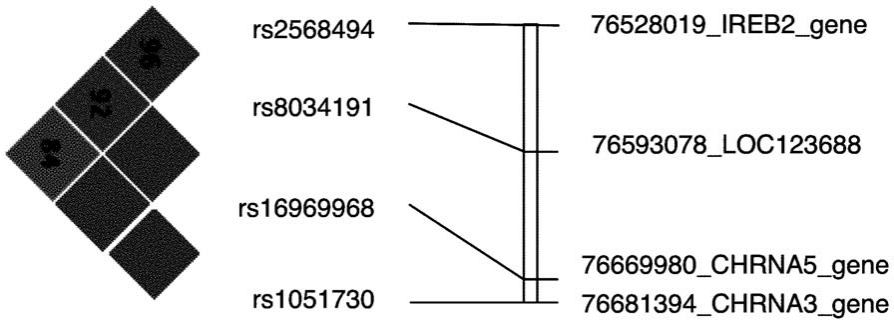
Linkage Disequilibrium Map for the COPD-associated variants in the 15q24/25 region. Red corresponds to r2≥0.8. Values for D′ are included in the text of boxes. The genomic positions were retrieved from the NCBI dbSNP identifier (NCBI Human genome Build 36 location). The CHRNA 5 gene variant, rs55853698, is not present in the HapMap [21].

The biology, by which rs1051730 and rs8034191 contribute to smoking-related disease phenotypes still remains unresolved. The rs1051730 SNP is a synonymous SNP located in exon 5 of the *CHRNA3* gene, which is in strong linkage disequilibrium with a non-synonymous variant rs16969968 in exon 5 of the *CHRNA5* gene [29] and with rs55853698 in the promoter region of CHRNA 5 (all pairwise r^2^>0,96; figure 1) [30]. The SNP rs16969968 results in an amino acid change (D398N) in the alpha5 receptor subunit protein and has been shown to affect receptor function [31]. The location of the rs55853698 variant makes it a candidate for affecting mRNA transcription. On the other hand, rs1051730 and rs8034191 are also strongly linked with rs2568494 in the IREB2 gene (r^2^ = 0.692 and r^2^ = 0.790, respectively; figure 1). Therefore genetic variation in the 15q24/25 region can also result in an altered function of the IREB2 gene. The finding of increased IREB2 protein and mRNA in lung-tissue samples from COPD subjects in comparison to controls supports a role of the IREB2 gene in COPD pathogenesis [32]. Additional functional analyses are therefore required to establish whether the CHRNA3, CHRNA5 or IREB2 genes are involved in COPD development. Moreover, not only genetic variation, but also the impact of epigenetic variation in these genes on the development of COPD should be investigated. For instance in lung cancer, it has been shown that the CHRNA3 gene is frequently hypermethylated in lung tumor tissue samples (in comparison with blood control samples) and that this hypermethylation, by inducing gene silencing, results in resistance against nicotine-induced apoptosis [33].

Regardless of the mechanisms by which *nAChR* variants predispose to an accelerated lung function decline in smokers, the identification of this genetic locus could have important clinical implications. First of all, this marker would contribute to the prospective identification of a subset of susceptible smokers at high risk for an accelerated loss of lung function. Secondly, since smoking cessation is the most effective way to reduce the rate of lung function decline, this information could be used to convince subjects to quit smoking before symptoms of COPD develop. Remarkably, we did not identify a significant association between genetic variation in the *nAChR* genes and FEV_1_ decline. However, as also suggested in another genetic association study on COPD-related phenotypes, it is likely that different genetic loci control FEV_1_ and FEV_1_/FVC, since both parameters reflect a different functional measure [34].

The major strength of the present study is that we were able to access a large number of apparently healthy, but heavy smokers, as well as a relatively large cohort of symptomatic COPD patients, including a significant number of patients with end-stage lung disease in need of lung transplantation. This enabled us to study the role of *nAChR* genetic variants in various stages of COPD severity. Furthermore, the large number of study participants allowed extensive corrections for potentially confounding factors such as pack-years and smoking status. Unfortunately, we were not able to correct for smoking intensity (for instance cigarettes smoked per day). Some other limitations need to be acknowledged as well. First, COPACETIC only recruited heavy smokers and we could therefore not assess the effects of the 15q24/25 locus in subjects being exposed to less nicotine. Second, our follow-up period was limited to three years and we only assessed lung function level at two different time points. Third, in the COPACETIC cohort only males were included. Future studies should include females to assess the interaction between rs1057130/rs8034191, smoking status and lung function decline since it is known that differences in smoking behavior and resulting lung function disturbances exist between the sexes. Lastly, we only had information on the smoking status at baseline. In theory some quitters could have started smoking again which could have influenced our results.

In conclusion, we have demonstrated that in a European population heavy smokers carrying homozygous at-risk alleles for rs1051730 and rs8034191 in the *nAChR* locus are characterized by an accelerated decline in lung function, possibly leading to an increased risk of developing severe COPD. We thus provide one of the first genetic markers predictive for lung function decline.

## Supporting Information

Material S1 Study population.
(DOC)

Material S2 Genotyping.
(DOC)

Material S3 Statistical analysis.
(DOC)
