# Supplementary material for: Variants in the 15q24/25 Locus Associate with Lung Function Decline in Active Smokers
Source: PLoS One. 2013 Jan 18;8(1):e53219. doi: 10.1371/journal.pone.0053219 (PMC3548843; doi:10.1371/journal.pone.0053219)
Supplement: Material S1 — Study population. (DOC) [file pone.0053219.s001.doc]

**Variants in the 15q24/25 locus associate with lung function decline in active smokers**

Firdaus A. A. Mohamed Hoesein^*^, Els Wauters^*^, Wim Janssens, Harry J. M. Groen, Joanna Smolonska, Cisca Wijmenga, Dirkje S. Postma, H. Marike. Boezen, Pim A. De Jong, Marc Decramer, Jan-Willem J. Lammers, Diether Lambrechts and Pieter Zanen

* These authors contributed equally to this work

**ONLINE SUPPLEMENT**

**Supplementary Material S1: STUDY POPULATION**

**COPACETIC STUDY POPULATION:** The **COPACETIC** cohort included 2,246 participants from the NELSON trial recruited by the University Medical Centers in Groningen and Utrecht. Of these 2,246 participants a random sample of 1,226 participants was selected for a genome-wide association study. The inclusion criteria of the NELSON trial are discussed in detail elsewhere . Briefly, the NELSON trial included current and former heavy smokers (pack-years <20) fit enough to undergo surgery. Those with moderate or bad self-reported health status were excluded. Only males were included based on the high risk to develop lung cancer or COPD as fewer women in the Dutch population have accumulated a long-term exposure to cigarettes compared to men. Baseline details on smoking habits were gathered through questionnaires which included questions about age at started smoking, duration of smoking, number of pack-years smoked, smoking status at enrolment (current or former smoker) and if applicable duration of cessation.

**Additional information regarding the COPACETIC genome-wide association study:** COPACETIC is an international and multicenter study that aims to elucidate the genetic basis of individual variance in the susceptibility to COPD and emphysema. In the discovery phase, 1078 participants with emphysema, 1036 participants with obstruction and 1677 non-obstructive, non-emphysema participants were included. These participants were recruited from the general population of 7 districts in the Netherlands: Groningen, Drenthe, Utrecht, Eemland, Midden-Nederland, Kennemerland and Amstelland-de Meerlanden . In particular, no COPACETIC subjects have been recruited in Belgium. All participants have been analyzed using Illumina’s genome-wide SNP arrays (Human610-Quad BeadChip) and a stringent quality control assessment on the data was performed. Since genome-wide findings in discovery cohorts are prone to high false-positive discovery rates, interesting SNPs are currently being replicated in >10,000 replication samples from COPACETIC. The replication phase is only expected to finish mid 2012.

Importantly, the 1,226 COPACETIC participants in the current study represent only a minority (32%) of the COPACETIC GWAS participants and even less (10%) of the combined COPACETIC discovery/replication cohort. In particular, these 1,226 subjects were selected because of the availability of lung function decline data, which are not available and will not be reported in the GWAS. The second study population of the current study, the LEUVEN cohort (which is described in detail below), is not part of the COPACETIC GWAS.

**LEUVEN STUDY POPULATION:** The **LEUVEN** cohort included 366 Belgian participants of the NELSON trial that were recruited from the general population of 14 municipalities around LEUVEN. These 366 population-based subjects were not previously diagnosed with COPD. In addition, 517 heavy smokers with symptoms compatible with COPD were included at the LEUVEN respiratory outpatient clinic. Of these 517 subjects, 123 were listed for lung transplantation because of disabling end-stage COPD . Inclusion criteria for these 883 LEUVEN participants were a smoking history of at least 15 pack-years, a minimal age of 50 years and the availability of a complete pulmonary function test. Subjects with a suspicion or diagnosis of asthma were excluded, as well as those with other respiratory diseases affecting pulmonary function. General listing criteria for lung transplantation were end-stage lung disease with serious repercussions on daily life activities, a life expectancy <18 months, <65 years of age and a smoke-free period of ≥6 months. Patients with COPD or emphysema were classified as having end-stage disease if they had a FEV_1_ <1 litre or an FEV_1_ % predicted <25, an arterial oxygen tension <60 mmHg and/or an arterial carbon dioxide tension >55 mmHg, failure of the right ventricle caused primarily by the respiratory disorder (cor pulmonale) or progressive deterioration of lung function despite optimal treatment (inclusive respiratory revalidation and oxygen therapy). Arterial blood gas values and echocardiographic measurements were only available for the minority of the total population (45% and 35%, respectively, and mainly consisting of the patients listed for transplantation) and were therefore not studied as separate phenotypes.

References

1. van Iersel CA, de Koning HJ, Draisma G, Mali WP, Scholten ET, et al. (2007) Risk-based selection from the general population in a screening trial: selection criteria, recruitment and power for the Dutch-Belgian randomised lung cancer multi-slice CT screening trial (NELSON). Int J Cancer 120: 868-874.

2. Janssens W, Bouillon R, Claes B, Carremans C, Lehouck A, et al. (2009) Vitamin D deficiency is highly prevalent in COPD and correlates with variants in the vitamin D-binding gene. Thorax 65: 215-220.
