# Supplementary material for: Variants in the 15q24/25 Locus Associate with Lung Function Decline in Active Smokers
Source: PLoS One. 2013 Jan 18;8(1):e53219. doi: 10.1371/journal.pone.0053219 (PMC3548843; doi:10.1371/journal.pone.0053219)
Supplement: Material S2 — Genotyping. (DOC) [file pone.0053219.s002.doc]

**Supplementary Material S2: GENOTYPING**

In COPACETIC, the rs1051730 and rs8034191 genotypes were extracted from Human610-Quad BeadChip data (Illumina Inc., San Diego, CA, USA). Quality control for these data included the removal of *i)* SNPs located in copy number variations, *ii)* SNPs out of Hardy-Weinberg equilibrium, *iii)* SNPs with a minor allele frequency <5%, and *iv)* SNPs with >5% of genotype data missing. Ethnic outliers were removed using principle component analysis as well as related individuals. From 620,901 SNPs genotyped, 522,902 SNPs passed the first round quality control, of which rs1051730 and rs8034191.

Genotyping in the LEUVEN cohort for the rs1051730 and rs8034191 SNPs was performed in a blinded manner using iPLEX technology on a MassARRAY Compact Analyser (Sequenom Inc., San Diego, CA, USA), as reported previously . Quality control was performed by genotyping 13 samples in duplicate, with a duplicate concordance of 100% between the samples.

References

1. Lambrechts D, Buysschaert I, Zanen P, Coolen J, Lays N, et al. (2010) The 15q24/25 susceptibility variant for lung cancer and chronic obstructive pulmonary disease is associated with emphysema. Am J Respir Crit Care Med 181: 486-493.
