# Supplementary material for: Variants in the 15q24/25 Locus Associate with Lung Function Decline in Active Smokers
Source: PLoS One. 2013 Jan 18;8(1):e53219. doi: 10.1371/journal.pone.0053219 (PMC3548843; doi:10.1371/journal.pone.0053219)
Supplement: Material S3 — Statistical analysis. (DOC) [file pone.0053219.s003.doc]

**Supplementary Material S3. STATISTICAL ANALYSIS**

**Power calculations**: we calculated the observed power to discover a significant association of genotypes, smoking status (current versus former) and the genotype*smoking status interaction term with lung function decline over a three-year follow-up period, as shown in Table S1.

Regarding the observed power for the association between decline in FEV1/FVC and the genotype*smoking status interaction, the established power of 81.9% and 88.0%, respectively for rs1051730 and rs8034191, indicates that there is a high probability that the absence of an association between genotype*former smoking status and lung function decline is a true negative finding. The same rationale applies to the observations for decline in MEF_50_.

In addition, to further demonstrate that the smaller sample size of the group of former smokers (n=479 compared with 753 current smokers) did not influence our findings, we stratified this group of ex-smokers according to rs1051730 genotypes and used the non-inferiority or equivalent approach, as described previously , to calculate a one-sided 95% confidence interval (CI) of the ratio of two means. We determined the two-sided 90% CI’s (which corresponds to a one one-sided 95% CI) for the differences between the ln-transformed FEV1/FVC values in the AA-, AG- and GG- genotypes of rs1051730. The width of those intervals is directly related to the power of the analysis. The largest difference in this stratified analysis appeared to be between AA- and AG-genotypes with a ratio of exp(0.010447)=101.105%. In other words, FEV1/FVC for AA-carriers at follow-up was 1.0111 times the value of AG-carriers. The 90% CI of that ratio ranges from exp(-0.006822) to exp(0.027715) or from 0.9932 to 1.0281. This means that, in the population of former smokers, the FEV1/FVC values will, with 90% certainty, be within the range of 99.320% to 102.810% of the FEV1/FVC value of AA-carriers at follow-up. The width of this interval is 101.105 – 99.320 = 1.785%. This means that the difference in FEV1/FVC between AA- and AG-carriers for rs1051730 will, with 95% certainty, not exceed 1.785% of the FEV1/FVC value at follow-up. Analogously, the FEV1/FVC difference between the GG and AG groups of rs8034191 will not exceed 0.8% of the actual mean FEV1/FVC in the GG group.

References

1. Schuirmann DJ (1987) A comparison of the two one-sided tests procedure and the power approach for assessing the equivalence of average bioavailability. J Pharmacokinet Biopharm 15: 657-680.

**Table S1.** The observed power for each test was calculated by use of the Generalized Linear Models method in SPSS (SPSS software version 18.0, Chicago, Illinois, USA).

|  | **Observed power** |
| --- | --- |
| **1. Decline in FEV1/FVC** |  |
| genotype |  |
| rs1051730 | 24% |
| rs8034191 | 15.2% |
| smoking status | ~100% |
| genotype * smoking status interaction |  |
| rs1051730 | 81.9% |
| rs8034191 | 88.0% |
| **2. Decline in FEV1** |  |
| Genotype |  |
| rs1051730 | 5.8% |
| rs8034191 | 7.8% |
| smoking status | ~91% |
| genotype * smoking status interaction | |
| rs1051730 | 33.4% |
| rs8034191 | 45% |
| **3. Decline in MEF50** |  |
| Genotype |  |
| rs1051730 | 15.3% |
| rs8034191 | 10.5% |
| smoking status | 95% |
| genotype * smoking status interaction | |
| rs1051730 | 59.3% |
| rs8034191 | 62.8% |
